# The effects of the pandemic on music teaching in schools in Quebec (Canada) in the spring and fall of 2020

**DOI:** 10.1177/02557614231157101

**Published:** 2023-03-03

**Authors:** Audrey-Kristel Barbeau, Hélène Boucher, Isabelle Héroux

**Affiliations:** Université du Québec à Montréal, Canada; Université du Québec à Montréal, Canada; Université du Québec à Montréal, Canada

**Keywords:** Canada, COVID-19, music teaching, online teaching, pandemic

## Abstract

COVID-19 containment measures brought many changes in our lives and forced teachers all around the world to adopt various new practices. Given its specific education requirements and numerous school boards, the province of Quebec, Canada, was chosen to study the effects of the pandemic on music teaching in schools in the spring and fall of 2020. An electronic survey was distributed, to which 517 elementary and high school music teachers responded. Teachers reported on the transformation of teaching modes from an exclusively in-person practice to an online or bimodal approach. Continuation and interruption of music programs varied greatly from school to school and, for those who were allowed musical activities, different protective health measures were implemented. Teachers working with large ensembles (e.g. band and orchestra) experienced more interruptions in their music programs. Teachers also reported how their planning was affected by the new modes of instruction, but no matter which modes were used, most of them experienced less motivation for teaching during the spring of 2020. In addition, they perceived that it was more motivating for students to receive an education in person. Finally, positive outcomes of the pandemic on education included the development of new skills in the use of digital resources and online teaching, as well as a renewed sense of solidarity between teachers.

The COVID-19 pandemic has created significant changes in our teaching practices all over the world, and music education researchers have started to report how the teaching of music has been affected. In England, Daubney and Fautley (2020) presented various issues: the shift toward online teaching and learning, curriculum and assessment, inadequate preparation of teachers, their new responsibilities and the lack of support received, and the training of future teachers. Furthermore, in Italy, Schiavio et al. (2021) documented the music learning experience of 20 conservatory students. Their results suggest that different approaches have been developed regarding practice and time management, modifying short- and long-term goals, increasing creative potential, and establishing new forms of peer interactions.

In Turkey, a study reported on the perception of teachers toward music education during the pandemic (Akyürek, 2020). Of the 46 participants, 89% had no prior preparation for distance education. When asked about their experience—the difficulties and the problems encountered—, teachers most frequently reported elements related to technologies. Low motivation was also an element that was mentioned frequently (Akyürek, 2020). Similarly, still in Turkey, Akarsu (2021) investigated the teaching of music in secondary schools. The findings indicate a lack of prior experience with online teaching and difficulty using the technology. The teachers felt it was “not suitable for music lessons,” and they reported low motivation from students (p. 170).

In the United States, Thornton (2020) offered a reflection on distance music education. She mentioned positive elements that emerged from this experience: an expanded space for musical creativity, an active and cooperative virtual community, and a reaction tinged with empathy, patience, and thoughtfulness toward the needs of the students. She also questioned the difficulty of creating a real musical experience in collective symbiosis, while noting that several students have made music by themselves.

Research in Sweden also explored cooperation through virtual communities. In fact, Thorgersen and Mars (2021) analyzed interactions on a music teachers’ Facebook group, and they found that it was an important medium for facilitating conversations about educational challenges and designing teaching situations that emerged with new teaching conditions. This became the space for material sharing and “letting off steam” (p. 231).

In Australia, a narrative reflection study reported on the work of two Australian music teachers—one in a teachers’ education program, the other in a primary school. On the themes of changes in practice and connection making, they list the positives and negatives of teaching music during COVID. Among the negative, they describe limited availability of material in students’ homes, difficulty in connecting with peers online, and highly time-consuming preparation for online teaching. On the positive side, they witnessed more space for reserved and shy students, better understanding of music learning from families, the possibility of recording classes for students to revise when needed, and an integration of technology into the music classroom (Joseph & Lennox, 2021).

While the pandemic impacted music teaching around the globe, no peer reviewed study in Canada was found. The only data available was found on the website of the Association of Canadian Choral Communities (2020). To discern how their members were affected by the pandemic, the organization conducted a survey in which 611 choral organizations participated, which represent 4% of Canadian choirs, the majority being amateur/non-professional (84%). Although only 14 groups were from educational institutions, their data provides important information about musical activities in Canada during COVID. They report that almost all organizations saw their activities disrupted which caused important financial losses, putting many choirs in a difficult position. These interruptions are also believed to have had an impact on the mental and physical well-being of the choristers, leading to a “profound sense of loss” of community. Even though virtual activities were put in place to maintain connections, they could not “replace the in-person experience of singing in a choir.”

Canada has advantageous economic means and is among the top ranked countries in the Program for International Student Assessment (PISA).^1^ However, its education system had not yet succeeded in integrating digital approaches in its pedagogical system at the time the pandemic was declared. It is thus interesting to observe how a world leader reacted to the challenges of the pandemic.

To better understand the consequences of the pandemic on music education in the schools in Quebec, Canada, we surveyed elementary and high school music teachers. The purpose of this research was to describe the effects of the pandemic on music teaching in schools in the spring and fall of 2020. Our research objectives were threefold:

RQ1: To describe changes in music teaching in elementary and high school during the pandemic.
RQ2: To report music teachers’ perceptions of planning, motivation, and students’ involvement during the pandemic.
RQ3: To analyze the use of, and training in, digital resources by music teachers during the pandemic in relation to the modes of teaching (in-person, online, and bimodal).

## Method

This project was approved by the ethics committee of the University of [redacted]. A survey, developed by the authors, was conducted in French, in the Province of Quebec, Canada. Due to the pandemic situation, we chose to develop an online survey, especially because it was a cost-sensitive method that allowed us to reach a large proportion of music teachers across the province (Minnaar & Heystek, 2013). It was pilot-tested beforehand in two phases: Phase one was done by a small sample of research assistants and served to verify that the paths to the conditional answers were complete. During phase two, a small sample of music teachers were asked to complete the survey to ensure the clarity of wordings and instructions (Cohen & Manion, 1998).

The survey, available on the Alchemer platform, was organized in three sections with a total of 101 questions; some were multiple-choice, while others were open-ended (see Supplemental Appendix 1 for full survey). In the first section, participants had to provide socio-demographic information about their professional situation, as well as any training they received in distance education and technological tools. In the second part, respondents were divided according to the teaching modes (in-person, online, and bimodal) that prevailed in their school when it reopened in spring 2020. The topics addressed concerned the availability and use of different digital resources in support of teaching and the application of different protective health measures. In addition, several questions were about modifications in their professional tasks, preparation for teaching, musical and pedagogical practices continued or ended, content taught and assessed, as well as motivation, and students’ participation. The third and final section used the same questions, this time focusing on the fall of 2020. An opening for participants to provide additional information and comments concluded the survey. The survey took approximately 10 to 20 minutes to complete.

Most of the open questions were oriented around targeted topics: technological tools used (*n* = 90), musical activities in spring (*n* = 123) and in autumn 2020 (*n* = 100), major changes between 2019 to 2020 and 2020 to 2021 teaching assignments (*n* = 57), and additional information (*n* = 104). We undertook content analysis with the Nvivo 12 software.

### Setting

In Canada, education is under provincial jurisdiction which leads to substantial differences in school organization and programs in the 10 provinces and 3 territories. In Quebec, education is provided primarily in French, the official language of the province, but also in English and some indigenous languages. There are 60 French school boards, 9 English school boards, and 2 school boards with special status across the province, for a total of 71 (Gouvernement du Québec, 2022). Whether the schools are public or private, they are all subject to the same organizational structure and program requirements (Ministère du travail, de l’emploi et de la solidarité sociale, 2022). Arts education is mandatory during elementary (grades 1–6) and high school (sec 1–5^2^). However, music is an option along with visual arts, dance, and drama. To become a music teacher, one has to complete a 4-year bachelor’s degree in music education to be considered a music specialist. In 2020 to 2021, there were 2,172 music specialists; 1,610 in primary schools and 562 in high schools (Institut de la statistique du Québec, 2022).

At the beginning of the pandemic, a full lock-down was declared in Quebec. All schools were closed on March 15 2020 to limit social contacts and slow transmission of the virus. Some jurisdictions with fewer COVID-19 cases were able to reopen schools with in-person teaching in April 2020, while others, including those in and around Montreal, had to do online teaching until the summer school break. This led to a diversification in teaching modes: online, in-person with safe distancing and health measures, and a combination of both (bimodal teaching). At the beginning of the school year in late August 2021, elementary and secondary schools were back to in-person teaching, but bimodal teaching was used when classes were temporarily closed due to COVID-19 outbreaks among students. Participants

The survey was conducted from September 21 to October 30, 2020. Using convenience and snowball sampling procedures (Cohen & Manion, 1998), it was distributed through music teachers’ local professional associations (via email through local representatives), various music teachers’ Facebook groups, and through the personal networks of the researchers. Out of a total possibility of 2,172 music teachers in Quebec, 517 responded, which resulted in a response rate of 23.8%. The participation rate was adequate for drawing valid statistical conclusions (Fosnacht et al., 2017). The margin of error of the survey was 4%, for a confidence index of 95%.

A total of 517 participants completed the first two sections of the survey about the 2020 spring situation, while 424 teachers completed the entire survey, also addressing questions related to autumn 2020. 88% of respondents worked in a public school and 85.5% within a regular music program. 66% taught in elementary school (children aged 5–12), 31% in high school (children aged 12–17), while 3% had a task involving both. Participants’ past teaching experience ranged from one year to over 41 years (*M* = 14.4, *SD* = 9.2).

Participants were primarily located in the Greater Montreal area: 22% in the Montreal areas and 22% in Montérégie (i.e. the South shore of Montreal). Some areas were underrepresented with regards to their real demographic weight. For instance, while Laval (located on the North shore of Montreal) has the third largest population in Quebec (Ministère de l’économie et de l’innovation, 2022), only 4% of music teachers located in that area participated in the survey (Boucher et al., 2022).

## Results

Results were analyzed using statistical testing, and descriptive statistics are provided. Parametric testing was conducted using *t*-test and ANOVA (Field, 2009; Olson, 1987). Among the non-parametric statistics used, Chi-square tests were selected for categorical data (Ferguson & Takane, 1989). When categorical variables were ordinal, Somers’ delta (or Somers’ *d*) was the preferred method to analyze results because it allowed us to make a distinction between the independent and dependant variables (Somers, 1962).

Not all respondents answered all the questions, as the survey was built using different paths with conditional questions. In addition, participants were sometimes allowed to provide more than one answer to the same question. This led to a variability in the number of respondents and answers in each section. To reflect this and ensure greater precision, we included the *n* in each table, and specified whether it represented answers or respondents. Additionally, when the number of answers or respondents was within the margin of error, we included them in the category *others.*

## Changes in music teaching

Teaching modes were impacted by the pandemic. As presented in Table 1, only 36% of the music teachers continued to teach in person during the first wave in the spring 2020, whereas 83% were back in person in the fall of 2020. More teachers used a combination of in-person and online (bimodal) teaching during the second wave; 16% in comparison to 6%. If we look at the overall number of teachers who stopped teaching in the spring, it represents more than a quarter of all teachers (28%).

**Table 1.** Teaching modes during spring and fall of 2020.

Teaching modes	Spring 2020 (*n* = 517^a^) (%)	Fall 2020 (*n* = 424^a^) (%)
School reopened and teaching continued in person	36	83
School did not reopen, and teaching continued online	30	1
School reopened and teaching continued in person AND online (bimodal)	6	16
Teachers were reassigned to other tasks	16	0
Teachers stopped teaching but were not reassigned to other duties	12	0

a *n* = respondents.

Many teachers saw their physical teaching space affected during the spring of 2020. The change that was mentioned most often was that music had to be taught in the children’s homeroom. Only 4% of the teachers who taught exclusively in person had to stop teaching music completely. The other changes that were reported included teaching from home (online), use of larger premises, and reorganization of the usual space (see Table 2). Fewer than 20% reported no changes.

**Table 2.** Changes in the physical space when using in-person teaching or bimodal teaching during the spring of 2020.

Changes in physical space	In-person (*n* = 240^a^) (%)	Bimodal (*n* = 32^a^) (%)
Music in the homeroom	36	31
Decrease in the number of students per class	24	16
None	17	19
Protective health measures applied	8	22
No music	4	0
Others	10	12

a *n* = respondents.

Many changes occurred in the tasks that teachers did during the spring and the fall of 2020. A notable change was the transition from high school to elementary school teaching. We also noticed that during the spring many teachers took the role of the generalist classroom teacher, substitute, or technological/administrative support, while changes in the fall tended to affect the workload, with some experiencing a reduction and others an increase (see Table 3).

**Table 3.** Changes in teaching tasks for the spring and fall of 2020.

Changes in teaching tasks	Spring 2020 (*n* = 127^a^) (%)	Fall 2020 (*n* = 53^a^) (%)
From high school to elementary	13	25
Became a generalist teacher	21	15
Technological or administrative support	20	0
Substitute teaching	11	0
Taught another subject	8	15
Did not teach	8	0
Individual student monitoring	5	0
Sick leave or maternity leave	5	0
Reduction of workload or early retirement	0	19
Increase in workload or return to full time	0	9
Change of student population	0	9
Others	10	11

a *n* = respondents.

The reasons teachers gave for these changes in their tasks were predominantly due to the end of their music program for protective health measures (25% in spring and 54% in fall). Reorganization of different types seemed to have played a role: lack of staff (15%), increase in the number of groups (10%), reassignment (10%), and class reorganization (11%). For the fall of 2020, personal decisions (15%) and lack of space (12%) also influenced these changes as presented in Table 4.

**Table 4.** Reasons for changes in teaching tasks for the spring and fall of 2020.

Reasons for changes in teaching tasks	Spring 2020 (*n* = 126^a^) (%)	Fall 2020 (*n* = 26^a^) (%)
Interruption of music program for protective health measures	25	54
Lack of staff	15	19
Classes reorganization	11	0
Increase in the number of groups	10	0
Reassignment	10	0
Personal decision	1	15
End of music program for lack of space	2	12
Illness or maternity leave	5	0
Others	21	0

a *n* = respondents.

Music teachers had to adapt their teaching activities to the limits imposed by the protective health measures in the spring of 2020. Their choices were particularly affected by the different modes of teaching they had to use (in-person, bimodal, or online) as shown in Table 5. Singing was mostly maintained or modified for those who remained on site while it was stopped or modified by those who were exclusively online. Recorder playing mostly ceased with 56% of all teaching modes not continuing this activity. Similarly, ukulele (64%) and guitar (60%) playing was curtailed, independently of the teaching modes. Most of the larger ensembles, regardless of teaching mode, had to discontinue their activities.

**Table 5.** Musical activities that were continued, modified, or stopped in the spring of 2020.

Musical activities	Spring 2020 in-person			Spring 2020^b^ bimodal			Spring 2020 online		
	Cont. (%)	Mod. (%)	Stop. (%)	Cont. (%)	Mod. (%)	Stop. (%)	Cont. (%)	Mod. (%)	Stop. (%)
Singing (*n* = 244^a^)	23	21	14	2	2	1	4	15	19
Orff ensemble (*n* = 192^a^)	6	16	40	0	2	2	1	2	33
Moving/dancing (*n* = 201^a^)	20	34	6	2	1	0	4	17	15
Recorder playing (*n* = 176^a^)	20	8	34	1	1	1	4	10	21
Musical games (*n* = 232^a^)	14	31	14	1	2	3	2	10	24
Ukulele (*n* = 110^a^)	12	17	33	0	1	4	1	5	27
Guitar (*n* = 112^a^)	16	9	26	1	2	3	1	11	31
Wind ensemble (*n* = 145^a^)	11	10	21	2	3	4	0	10	39
Stage band (*n* = 70^a^)	4	14	17	1	0	10	0	4	49
String orchestra (*n* = 33^a^)	15	9	27	0	0	3	3	9	33
Musicals (*n* = 33^a^)	0	6	45	0	3	6	0	6	33
Band (pop music) (*n* = 51^a^)	6	10	27	2	2	10	0	4	39
Symphony orchestra (*n* = 19^a^)	0	11	32	0	0	5	0	11	42
Percussions (djembe and drum lines) (*n* = 174^a^)	9	29	28	1	2	1	0	11	20

a *n* = answers

b The number of teachers who taught in a bimodal format (in-person and online) represents only 6% of the total sample, as presented in Table 1. Therefore, these numbers are to be interpreted with caution.

Table 6 reports the situation for the Fall of 2020. Since the survey was administered at the very beginning of the fall semester, most schools were back to on-site teaching. Singing was mostly continued or modified (89%), while 67% of answers indicated that Musicals were stopped. This is a noticeable paradox, as these two activities focus mostly on the use of the voice. It is worth nothing that Musicals tend to be part of after school programs which were forbidden by the government during the spring of 2020.

**Table 6.** Musical activities that were continued, modified, or stopped in the fall of 2020.

Musical activities	Fall 2020 in-person		
	Cont. (%)	Mod. (%)	Stop. (%)
Singing (*n* = 333^a^)	47	42	11
Orff ensemble (*n* = 265^a^)	18	53	29
Moving/dancing (*n* = 262^a^)	51	45	4
Recorder playing (*n* = 250^a^)	51	26	23
Musical games (*n* = 311^a^)	38	51	11
Ukulele (*n* = 136^a^)	28	39	33
Guitar (*n* = 149^a^)	36	34	30
Wind ensemble (*n* = 162^a^)	27	46	28
Stage band (*n* = 70^a^)	16	29	56
String orchestra (*n* = 25^a^)	40	28	32
Musicals (*n* = 39^a^)	5	28	67
Band (pop music) (*n* = 62^a^)	21	34	45
Symphony orchestra (*n* = 12^a^)	8	33	58
Percussions (djembe and drum lines) (*n* = 230^a^)	21	58	21

a *n* = answers.

## Music teachers’ perceptions of planning, motivation, and students’ involvement

We asked participants if they had been able to follow their regular planning during the pandemic by answering questions on a scale ranging from not at all (0) to fully (3). We included only teachers who taught exclusively in one level of education, be it elementary (*n* = 211) or high school (*n* = 106). No statistical difference was found. Both yielded means below 1, indicating that most were only slightly able (if at all) to follow their pre-pandemic planning. We then compared how the modes of teaching (in-person or online) could affect music teachers’ perception of planning. There was a statistically significant difference between in-person teaching (*M* = 0.84, *SD* = 0.78) and online teaching (*M* = 0.42, *SD* = 0.60). Online teaching required more adaptation to the teachers’ planning than in-person teaching, *t*(305) = 5.34, *p* < .001, *r* = .29.

Teachers assessed their personal motivation toward teaching during spring and fall of 2020. At the beginning of the pandemic, 61% reported feeling less motivated, 30% as motivated as usual, and 9% more motivated (*n* = 411). In the fall, 18% reported feeling less motivated, 42% as motivated as usual, and 40% more motivated (*n* = 381).

Students’ motivation was explored through teachers’ perceptions. During the spring of 2020, 63% of teachers reported less motivation from their students, 26% no change in motivation and 11% more motivation (*n* = 328). Somers’ *d* was run to determine the association between the teachers’ perception of their students’ motivation and their own motivation. There was a positive association between teachers’ and students’ motivation, which was statistically significant (*d* = 0.298, *p* < .001). Among the more motivated teachers, they perceived that almost half of their students (48%) were highly motivated, almost a fifth (19%) were as motivated as usual and a third (33%) were less motivated. Among the teachers who kept the same level of motivation, they perceived that half of their students (50%) were less motivated, 39% were as motivated as usual and 11% were more motivated. The teachers who reported feeling less motivated perceived that most of their students (74%) were less motivated, whereas a fifth (21%) were considered as motivated as usual, and very few (5%) more motivated. These results show that teachers who were less motivated were more likely to perceive that their students were also less motivated, as compared to music teachers who reported normal or higher levels of self-motivation.

We also analyzed whether music teachers’ motivation was affected by the modes of teaching (in-person or online). Among the 124 teachers doing in-person activities during the spring, 57% reported being less motivated, 35% as motivated as usual, and 8% more motivated. Among the 111 teachers doing online activities, 68% reported being less motivated, 27% as motivated as usual, and 5% more motivated. No significant association was found between the modes of teaching (in-person or online) and teachers’ motivation χ^2^(2) = 2.71, *p* > .05. This means that teachers reported lower motivation when they started teaching again during the pandemic, no matter whether it was in-person or online. Additionally, we explored whether the modes of teaching (in person or online) could influence students’ motivation as perceived by music teachers (*n* = 306). A significant association was found χ^2^(2) = 85.79, *p* < .001. Post hoc comparisons for modes of teaching by student motivation showed that among the highly motivated students, more were being taught in-person (94.4% vs. 5.6% online), which was similar with students who maintained their usual level of motivation (86.3% in-person vs. 13.8% online). Among less motivated students, more were being taught online (65.3% vs. 34.7% in person).

We investigated teachers’ perceptions of online participation in elementary and high school during the spring of 2020. Music teachers (*n* = 139) indicated whether their students showed low, moderate or high participation during online activities. Elementary teachers (*n* = 69) reported that 61% of their students demonstrated low participation, 20% moderate participation, and 19% high participation. Results were more balanced among high school teachers (*n* = 70), who reported that 44% of their students demonstrated low participation, 20% moderate participation, and 36% high participation. However, no significant association was found between the levels of education teachers taught in (elementary or high school) and students’ participation rates in learning activities, χ^2^(4) = 6.82, *p* > .05. These results seem to indicate that participation in online activities was similarly low for both elementary and high school students.

## Use of, and training in, digital resources

Music teachers’ technological competencies were required during COVID-19. The levels of education in which music teachers were involved (elementary, high school, or both) were compared with self-perceived technological competencies, assessed on a scale ranging from 0 (very low) to 4 (very high). No significant effect was found among elementary school teachers (*n* = 341, *M* = 2.31, *SD* = 0.83), high school teachers (*n* = 159, *M* = 2.43, *SD* = 0.74), and teachers involved in both levels (*n* = 17, *M* = 2.59, *SD* = 0.94), *F*(2, 514) = 2.06, *p* > .05.

We explored whether years of experience had an impact on self-perceived technological competencies. We found a significant, though relatively small, effect for years of experience in music teaching and self-perceived technological competencies, Welch’s *F*(2, 219) = 3.86, *p* < .005, ω = .03. Tukey’s HSD post-hoc analysis revealed that music teachers with 5 years of experience or less reported higher degrees of self-perceived technological competencies (*M* = 2.54, *SD* = 0.73) than teachers with more than 25 years of experience (*M* = 2.03, *SD* = 0.94). A significant difference was also found between teachers with 6 to 10 years of experience (*M* = 2.41, *SD* = 0.84) and those with over 25 years of experience. No other significant differences were found among teachers.

When asked if they had done any training to improve their technological competencies during the spring of 2020, 68% answered positively. The majority of these were offered by the school boards (45%) and individuals (17%) as presented in Table 7. They were followed autonomously (31%) or given by a facilitator (28%). It is interesting to note that 25% reported idea sharing (informal meetings with peers on Zoom or Facebook) as a form of professional development (see Table 8). As presented in Table 9, these training sessions focused mostly on using online platforms to teach (31%) and on how to use applications and software (19%).

**Table 7.** Professional development providers who offered trainings during the spring of 2020.

Professional development providers	Spring 2020 (*n* = 598^a^) (%)
School board	45
Individuals	17
Professional association	14
University	14
Others Educational businesses	10

a *n* = answers.

**Table 8.** Type of professional development taken during the spring of 2020.

Types of professional development	Spring 2020 (*n* = 845^a^) (%)
Autonomously (online tutorials)	31
With a facilitator (live online)	28
Idea sharing (informal meeting with peers on Zoom or on Facebook)	25
Community of practice (organized work group with peers)	16

a *n* = answers.

**Table 9.** Topics of the professional development taken during the spring of 2020.

Topics of professional development	Spring 2020 (*n* = 909^a^) (%)
Online platforms	31
Applications or software	19
Online educational activity design tools	14
Access to ready to use educational material	12
Online tools for facilitating educational activities	10
Hygiene and protective health measures	10
Others	1

a *n* = answers.

When forced to use online teaching, most music teachers sought assistance from their fellow music teachers or consulted specialized websites to find help and support as presented in Table 10.

**Table 10.** Resources toward which teachers sought assistance for online teaching.

Resources for assistance with online teaching	Bimodal (*n* = 42^a^) (%)	Online (*n* = 329^a^) (%)
Music teachers	26	30
Websites	24	20
Pedagogical advisors	14	9
Professional associations	10	6
Ministry of education’s publications	7	9
None	7	5
Provincial TV channel (pedagogical videos)	5	9
Generalist teachers	5	5
Others	2	7

a *n* = answers.

## Discussion

This article reports how music teachers experienced changes in their professional lives in relation to the pandemic in the spring and fall of 2020. They reported on the transformation of teaching modalities, from an exclusively in-person practice to an online or bimodal approach. Continuation and interruption of music programs varied greatly from school to school and, for those who were allowed musical activities, different protective health measures were implemented. Teachers working with large ensembles (e.g. band and orchestra) experienced more interruptions in their music programs. They also reported how their planning was affected by the new modes of instruction, but regardless of the mode, most experienced less motivation for teaching during the spring of 2020. In addition, they perceived that it was more motivating for students to receive an education in person. Finally, positive outcomes of the pandemic are the development of new skills in the use of digital resources and online teaching as well as a revitalized peer support system.

As indicated above, the survey included multiple-choice answers and several open-ended questions to allow participants to complement their answers. We analyzed the latter, as well as the final comment box, to deepen our understanding of the results regarding our three research objectives.

In relation to our first objective, it is important to know that school administrations reacted differently to the pandemic, as illustrated by one of the 104 participants who provided additional information: “In two of my four schools, I have no access or partial access to the music rooms, which becomes very complex for implementing learning activities” (P481). This might explain the variability in our results about physical space, reasons for changes in tasks, and impacts on musical activities that were continued.

While some administrations were supportive and purchased more instruments, others feared virus transmission associated with singing, wind playing, and the sharing of equipment between students. In reaction, some proposed special measures to lower the risks or simply stopped music classes. Some music teachers who were permitted to teach in their usual classrooms had to disinfect chairs, floors, and instruments between each group. The situation was particularly demanding in elementary schools in which teachers used many instruments:

There are not enough instruments available for me to make dedicated bins for each group. Musical games and movement are limited now. For prevention purposes, singing and recorder playing must follow additional rules. Some instruments simply cannot be disinfected with the products provided by the schools. They must be left in quarantine for 72 hours before being used with another group. This is a complex logistic, especially when teaching in multiple schools (P94).

In addition, the protective health measures were interpreted differently by the homeroom teachers: “In some classes, children are forced by their teachers to learn in the cold (all windows and doors are completely open). Therefore, the children wear their coats, hats, gloves and are freezing. COVID or pneumonia! In these conditions, the children can’t sing” (P459). On the other hand, it is likely that the schools that were not overly affected by the physical changes and protective health measures were in areas less affected by COVID, with fewer students and with facilities that allowed for good distancing of students. “[I had the] encouragement of my direction to teach as closely as possible to my usual practice without changing it too much. We follow the public health regulations to the letter. But I kept my classroom, recorder playing, singing. . .(P452).”

In line with public health and government guidelines (INSPQ, 2021), when schools reopened (spring and fall), a two-metre minimal space between students was mandatory at all times. In addition, in fall 2020, the “classroom bubbles” were imposed, meaning that the same children would interact together throughout each day, having all their classes together, eating together, and interacting on the playground amongst themselves only. This made it impossible to have students from separate groups be part of a chosen activity, such as music. “I had to stop my choir because the children come from different classes, and we have to respect the concept of the classroom bubbles" (P324). The complexity of the situation may explain why some school administrations simply stopped music education. It seems that music instruction was interrupted more often in high schools, where groups of students do not remain consistent from one subject to the next. This may explain in part the large transfer of teachers from high school to elementary school in the fall of 2020.

Moreover, concerns about transmission of COVID and public health guidelines had an impact on activities that were continued, modified, or stopped in the spring or fall of 2020. It is obvious from Table 5 that large musical ensembles suffered the most from the pandemic. “The instrument [playing], concert bands and musicals are on pause [since spring]. I hope to be able to restart the instruments next week [in autumn], I have given up hope on the rest [all the other musical activities]” (P352). These large ensembles are more common in high schools and were also more affected by the interruption of music classes in the fall of 2020 (Table 6). Additionally, extra-curricular activities, often including large ensembles and musicals, were forbidden by the government during the spring of 2020.

Our second research objective was focusing on music teachers’ perceptions in relation to planning, motivation and perceived students’ involvement during the pandemic. Interestingly, our data shows no significant differences between elementary or high school in relation to planning. As expected, online teaching did not allow teachers to follow their initial planning and required more work. This echoes Joseph and Lennox’s testimony (2021) about the time-consuming preparation needed for online teaching. Teachers also reported being less motivated in the first wave of the pandemic in the spring (61%) than in the fall. Surprisingly, we did not find a significant difference in teachers’ motivation related to the mode of instruction. One may hypothesize that all the changes required to go back to in-person teaching (safe distancing, mask wearing, and disinfection), as well as the uncertainties associated with the pandemic, took a toll on people’s motivation.

On the other hand, teachers perceived that it was more motivating for students to receive in-person rather than online teaching. This result is in line with that of Akarsu (2021), who reported low motivation from the students. In addition, teachers who were less motivated tended to perceive less motivation in their students. We found nothing in our analysis to explain the perceived lack of motivation among teachers during the pandemic in terms of planning, switching to online teaching, levels of education which they taught, or years of experience. It is therefore possible that the general decrease in motivation was created by the anxiety-inducing climate of the pandemic. “Everything is stressful, difficult. (. . .) A colleague has been absent for over a month, she has contracted COVID and is very ill. It’s discouraging. I understand that we need to keep the school open, the students need it. But the risk is there and that alone is stressful and scary” (P545).

In relation to our third research objective, we found that one of the positive outcomes of the pandemic on education was the development of skills for using digital resources. Indeed, 68% of our respondents reported taking part in training in relation to their employment during the spring of 2020, and these sessions focused mostly on using online platforms, applications, and software. Akyürek (2020), who reported that 89% of their participants had no prior preparation for distance education before the pandemic, also found that properly mastering digital distance education was difficult for teachers. From our data, we see that our participants were active in overcoming this challenge, and one could hypothesize that these new skills are likely to impact their teaching for the years to come. Moreover, it is worth noting that informal meetings with peers through online platform were perceived by 25% of our respondents as a form of professional development. This reinforces the perception that the pandemic offered the possibility of developing an active and cooperative virtual community among music teachers (Thorgersen & Mars, 2021; Thornton, 2020). As a participant summed up: “A big thank you to my music colleagues who have been tirelessly sharing their knowledge and discovery on Facebook. They were by far the most efficient and knowledgeable” (P155).

Additionally, before being able to teach online, teachers needed to learn how to use unfamiliar digital resources and platforms. Since such training was exclusively available online, they had to become familiar with the technology in order to even follow training about the specific resource. “. . .the training sessions only dealt with the use of tools (e.g. Teams, Mosaik), the definition of the word *presential* [face-to-face] and the difference between the words *synchronous* and *asynchronous*. We had a long way to go!!!! (P127).” The learning was therefore technocentric (Couture 2020), centered on the technology rather than on the pedagogy.

The first training sessions available were provided by the school boards to help as many teachers as possible maneuver online platforms (i.e. TEAMS, Zoom). Unfortunately, these were not designed to support the specific needs of the music specialists. This might explain the results presented in Table 8 in which music teachers turned to self-learning and peers to find solutions to their disciplinary technological needs. Like Thorgersen and Mars (2021), we found that colleagues, through Facebook, played an important support role: “A big thank you to my music colleagues who have been tirelessly sharing their knowledge and discovery on the Facebook pages. They were by far the most efficient and knowledgeable. I discovered a series of applications, and I was able to quickly get relevant, diversified, and not biased answers” (P155). Overall, although teaching with the digital tools was challenging, one of the positive outcomes of the pandemic is the renewed support system between music teachers.

## Limits and recommendations

Although this survey had a high response rate which brings reliability to our results, a limit of this type of data collection is that participants self-reported their views and observations. Moreover, the data was collected when they were still living the consequences of teaching during the pandemic. Another limit to our study is the structure of the survey that limited the number of statistical analyses. Indeed, many questions yielded categorical variables, limiting the parametric analyses available. Therefore, several of our results use descriptive statistics only. Although this provides an interesting portrait of the situation, it limits our understanding of the relationships between variables. Additionally, participants were recruited and the survey was distributed exclusively online. This brings a bias in which only the people who were already interacting with technology were invited to participate.

## Implications

The pandemic forced music educators to develop their digital skills. However, from our data, we see that this was limited to learning to use various technologies. To become meaningful, a shift still needs to happen from a technocentric approach to a proper digital pedagogy in which the teaching and learning become the centerpiece, supported by technology rather than being centered on it. These findings reveal a crucial need for proper training and the development of teaching resources within a digital framework. Researchers and practitioners now need to work together to support initial training and professional development of music educators. This study also provides information on the role that Canada could play as a leader in the world of education in ensuring that these new tools are made available to less fortunate communities. Some questions remain unanswered, however. Will teachers maintain some of the new strategies learned during COVID or will they retreat to their familiar pre-COVID ways of teaching? Will the schools and governments lend financial and ideological support to the use of digital resources and know-how? Will the technology develop to allow the proper quality of synchronicity and sound that music teaching requires to support live music? It will therefore be important for researchers to investigate how this will evolve over time.

## Supplemental Material

sj-pdf-1-ijm-10.1177_02557614231157101 – Supplemental material for The effects of the pandemic on music teaching in schools in Quebec (Canada) in the spring and fall of 2020 Click here for additional data file. Supplemental material, sj-pdf-1-ijm-10.1177_02557614231157101 for The effects of the pandemic on music teaching in schools in Quebec (Canada) in the spring and fall of 2020 by Audrey-Kristel Barbeau, Hélène Boucher and Isabelle Héroux in International Journal of Music Education

## Notes

1. On an international level, Canada has performed very well in all of the PISA assessments. For example, in the 2018 assessment, Canadian 15-year-olds placed well above the OECD average, and were among the top performers in reading. Of the 79 countries and economies that participated in the assessment, only three—Beijing, Shanghai, Jiangsu, Zhejiang (B-S-J-Z; China), Singapore, and Macao (China)—outperformed Canada (Council of Ministers of Education, Canada, 2022).

2. This corresponds to grades 7 to 11.
