# Supplementary material for: The effects of the pandemic on music teaching in schools in Quebec (Canada) in the spring and fall of 2020
Source: Int J Music Educ. 2023 Mar 3:02557614231157101. doi: 10.1177/02557614231157101 (PMC9988612; doi:10.1177/02557614231157101)
Supplement: sj-pdf-1-ijm-10.1177_02557614231157101 – Supplemental material for The effects of the pandemic on music teaching in schools in Quebec (Canada) in the spring and fall of 2020 [file sj-pdf-1-ijm-10.1177_02557614231157101.pdf]

Musique en temps de pandémie

Formulaire de consentement éthique pour la participation au sondage (obligatoire pour avoir accès au sondage)

ID 342

1. Merci de lire le formulaire de consentement éthique en cliquant sur l'hyperlien bleu en bas de cette page et de cocher Oui pour participer au sondage.

Vous devez aussi choisir une des options a) ou b) pour l'utilisation de vos données qui sont toujours anonymes.

Vos réponses à ce sondage sont anonymes. Nous aimerions les utiliser dans d'autres projets de recherche similaires. Vous êtes libre de refuser cette utilisation secondaire.

*

- a) J'accepte que mes données anonymes puissent être utilisées dans d'autres projets de recherche
- b) Je refuse que mes données anonymes puissent être utilisées dans d'autres projets de recherche

SECTION A Pour mieux connaitre votre situation

LOGIC Hidden unless: QUESTION NOT FOUND! is one of the following answers [NO OPTIONS SET]

ID 2

2. Dans quelle région administrative êtes-vous? *

- Abitibi-Témiscamingue
- Bas-Saint-Laurent
- Capitale-Nationale
- Centre-du-Québec
- Chaudière-Appalaches
- Côte-Nord
- Estrie
- Gaspésie–Îles-de-la-Madeleine
- Lanaudière
- Laurentides
- Laval
- Mauricie
- Montérégie
- Montréal
- Nord-du-Québec
- Outaouais
- Saguenay–Lac-Saint-Jean

ID 344

3. Quel type d'école? *

- Privée
- Publique
- Les deux
- Autres

ID 343

4. Quel type de programme? *

- [ ] Programme régulier
- [ ] Option
- [ ] Concentration ou musique-étude
- [ ] Other - Write In

ID 4

5. À quel niveau enseigniez-vous en février 2020? *

- ( ) Primaire
- ( ) Secondaire
- ( ) Primaire et secondaire

ID 5

6. À quel niveau enseignez-vous en septembre 2020? *

- ( ) Primaire
- ( ) Secondaire
- ( ) Primaire et secondaire

ID 6

7. Combien d'années d'expérience avez-vous dans l'enseignement scolaire (ex. 2)? *

ID 7

8. Comment évaluez-vous vos compétences en technologie numérique ? *

- Très faibles
- Faibles
- Moyennes
- Élevées
- Très élevées

ID 8

9. Comment évaluez-vous votre intérêt général envers les technologies numériques? *

- Très faible
- Faible
- Moyenne
- Élevée
- Très élevée

ID 9

10. Comment évaluez-vous la qualité de votre connexion internet à la maison? *

- Aucune
- Médiocre
- Acceptable
- Élevée
- Très élevée

SECTION B Suite au début de la pandémie

LOGIC Show/hide trigger exists.

ID 10

11. Avez-vous suivi des formations (avec un formateur ou de manière autonome) en lien avec votre travail ? *

- Oui
- Non

LOGIC Show/hide trigger exists. Hidden unless: #11 Question "Avez-vous suivi des formations (avec un formateur ou de manière autonome) en lien avec votre travail ?" is one of the following answers ("Oui")

ID 11

12. Par qui étaient offertes les formations que vous avez suivis en lien avec votre travail ? *

- [ ] École, Commissions scolaires, Conseillers pédagogiques
- [ ] Associations professionnelles
- [ ] Entreprises privées
- [ ] Initiatives de particuliers
- [ ] Universités
- [ ] Autre, merci de préciser

*

LOGIC Hidden unless: #12 Question "Par qui étaient offertes les formations que vous avez suivis en lien avec votre travail ?" is one of the following answers ("Associations professionnelles")

ID 257

13. Quelle association professionnelle donnait les formations que vous avez suivies (choix multiples possibles) ? *

- [ ] FAMEQ
- [ ] Orff-Québec
- [ ] Kodaly-Québec
- [ ] FOSHQ
- [ ] Autre, merci de préciser

LOGIC Hidden unless: #12 Question "Par qui étaient offertes les formations que vous avez suivis en lien avec votre travail ?" is one of the following answers ("Entreprises privées")

ID 258

14. Quelle entreprise donnait la ou les formations que vous avez suivies (plusieurs réponses possibles) ? *

LOGIC Hidden unless: #12 Question "Par qui étaient offertes les formations que vous avez suivis en lien avec votre travail ?" is one of the following answers ("Initiatives de particuliers")

ID 259

15. De quelles initiatives de particuliers avez-vous bénéficiées? *

LOGIC Hidden unless: #12 Question "Par qui étaient offertes les formations que vous avez suivis en lien avec votre travail ?" is one of the following answers ("Universités")

ID 260

16. Quelle université donnait là où les formations que vous avez suivies (plusieurs choix possibles) ? *

- ☐ Université du Québec
- ☐ TÉLUQ
- ☐ Université de Montréal
- ☐ Université McGill
- ☐ Université Laval
- ☐ Université Concordia
- ☐ Autre, merci de préciser *

LOGIC Hidden unless: #12 Question "Par qui étaient offertes les formations que vous avez suivis en lien avec votre travail ?" is one of the following answers ("Autre, merci de préciser")

ID 261

17. Qui donnait les formations que vous avez suivies? *

LOGIC Hidden unless: #11 Question "Avez-vous suivi des formations (avec un formateur ou de manière autonome) en lien avec votre travail ?" is one of the following answers ("Oui")

ID 288

18. Quel type de formation avez-vous suivi en lien avec votre travail (plusieurs choix possibles) ?

- [ ] En présence d’un formateur en ligne et en direct
- [ ] De manière autonome (ex. lire, regarder des tutoriels sur You Tube, formation asynchrone)
- [ ] Partage d’idée (rencontre informelle avec des pairs sur Zoom ou sur Facebook)
- [ ] Communauté de pratique (groupe organisé de travail avec des pairs ex. dans l'école, commission scolaire/centre de services)
- [ ] Autre, merci de préciser

*

LOGIC Show/hide trigger exists. Hidden unless: #11 Question "Avez-vous suivi des formations (avec un formateur ou de manière autonome) en lien avec votre travail ?" is one of the following answers ("Oui")

ID 12

19. Quel(s) sujet(s) étaient traités dans la ou les formations que vous avez suivies (plusieurs choix possibles) ? *

- ☐ Appropriation des plateformes en ligne
- ☐ Outils de conception d'activités pédagogiques
- ☐ Outils d'animation d'activités pédagogiques
- ☐ Accès à du matériel pédagogique clef en main
- ☐ Hygiène et mesures sanitaires
- ☐ Applications ou logiciels
- ☐ Autre, merci de préciser

*

LOGIC Hidden unless: #19 Question "Quel(s) sujet(s) étaient traités dans la ou les formations que vous avez suivies (plusieurs choix possibles) ?" is one of the following answers ("Appropriation des plateformes en ligne")

ID 262

20. Sur quelle plateforme en ligne portait votre formation? *

- ☐ Teams
- ☐ Zoom
- ☐ Skype
- ☐ Google Classrooms
- ☐ Autre

*

LOGIC Hidden unless: #19 Question "Quel(s) sujet(s) étaient traités dans la ou les formations que vous avez suivies (plusieurs choix possibles) ?" is one of the following answers ("Outils de conception d'activités pédagogiques")

ID 263

21. Sur quel(s) outil(s) de conception portait votre formation? *

- [ ] Genial.ly
- [ ] Kahoot!
- [ ] Smore
- [ ] Padlet
- [ ] Wakelet
- [ ] Autre

*

LOGIC Hidden unless: #19 Question "Quel(s) sujet(s) étaient traités dans la ou les formations que vous avez suivies (plusieurs choix possibles) ?" is one of the following answers ("Outils d'animation d'activités pédagogiques")

ID 264

22. Sur quel(s) outil(s) d'animation pédagogique portait votre formation? *

- [ ] Applications
- [ ] Logiciels
- [ ] Site Web
- [ ] Plateforme d'enseignement
- [ ] Ed Puzzle
- [ ] Map interactive
- [ ] Autre

*

LOGIC Hidden unless: #19 Question "Quel(s) sujet(s) étaient traités dans la ou les formations que vous avez suivies (plusieurs choix possibles) ?" is one of the following answers ("Accès à du matériel pédagogique clef en main")

ID 265

23. De quel type de matériel clef en main avez-vous utilisé (plusieurs réponses possibles)? *

- [ ] Payant, merci de préciser
- [ ] Gratuit, merci de préciser
- [ ] Privé (ex. intranet de l'école, obligation d'être membre). Merci de préciser
- [ ] Autre, merci de préciser

*

LOGIC Hidden unless: #19 Question "Quel(s) sujet(s) étaient traités dans la ou les formations que vous avez suivies (plusieurs choix possibles) ?" is one of the following answers ("Applications ou logiciels")

ID 266

24. Sur quel(s) application(s) ou logiciel(s) portaient les formations (plusieurs choix possibles) ? *

- [ ] Garage Band
- [ ] Audacity
- [ ] Abbleton
- [ ] Prezi
- [ ] PowerPoint
- [ ] Autre

*

LOGIC Hidden unless: #19 Question "Quel(s) sujet(s) étaient traités dans la ou les formations que vous avez suivies (plusieurs choix possibles) ?" is one of the following answers ("Autre, merci de préciser")

ID 267

25. Pouvez-vous préciser quel(s) était les sujets des formations que vous avez suivies? *

ID 225

26. Vers lesquelles de ces ressources vous-êtes-vous tournées pour vous aider à enseigner pendant la pandémie (plusieurs choix possibles) ? *

- ☐ Pistes pédagogiques du Ministère de l'éducation produites pendant la pandémie
- ☐ Capsules télévisuelles produites par Télé-Québec
- ☐ Conseillers pédagogiques assignés à votre école
- ☐ Enseignants généralistes
- ☐ Autres enseignants en musique
- ☐ Sites web commerciaux
- ☐ Sites web d'associations professionnelles
- ☐ Autres (précisez) *
- ☐ Aucune

LOGIC Show/hide trigger exists.

ID 15

27. Quelles sont les ressources pédagogiques que vous avez utilisées pendant la pandémie ? *

- [ ] Plateformes numériques
- [ ] Outils de conception d'activités pédagogiques
- [ ] Outils d'animation d'activités pédagogiques
- [ ] Matériel pédagogique clef en main
- [ ] Applications ou logiciels
- [ ] Hygiène et mesures sanitaires
- [ ] Autre (précisez) ______ *
- [ ] Aucune ______

LOGIC Hidden unless: #27 Question "Quelles sont les ressources pédagogiques que vous avez utilisées pendant la pandémie ?" is one of the following answers ("Plateformes numériques")

ID 272

28. Quelles plateformes avez-vous utilisé(s)? *

- [ ] Teams
- [ ] Zoom
- [ ] Skype
- [ ] Google Classroom
- [ ] Autre ______ *

LOGIC Hidden unless: #27 Question "Quelles sont les ressources pédagogiques que vous avez utilisées pendant la pandémie ?" is one of the following answers ("Outils de conception d'activités pédagogiques")

ID 273

29. Quel(s) outil(s) de conception d'activités pédagogiques avez-vous utilisé(s)? *

- [ ] Genial.ly
- [ ] Kahoot!
- [ ] Prezi
- [ ] Smore
- [ ] Padlet
- [ ] Wakelet
- [ ] Autre

*

LOGIC Hidden unless: #27 Question "Quelles sont les ressources pédagogiques que vous avez utilisées pendant la pandémie ?" is one of the following answers ("Outils d'animation d'activités pédagogiques")

ID 274

30. Quel(s) outil(s) d'animation d'activités pédagogiques avez-vous utilisé(s)? *

- [ ] Application
- [ ] Logiciel
- [ ] Site Web
- [ ] Plateforme d'enseignement
- [ ] Autre

*

LOGIC Hidden unless: #27 Question "Quelles sont les ressources pédagogiques que vous avez utilisées pendant la pandémie ?" is one of the following answers ("Matériel pédagogique clef en main")

ID 275

31. Quel type de matériel clef en main d'activités pédagogiques avez-vous utilisé(s)? *

- [ ] Gratuit, merci de préciser
- [ ] Payant, merci de préciser
- [ ] Privé (ex. site intranet école ou avec obligation d'être membre). Merci de préciser.
- [ ] Autre, merci de préciser

*

LOGIC Hidden unless: #27 Question "Quelles sont les ressources pédagogiques que vous avez utilisées pendant la pandémie ?" is one of the following answers ("Applications ou logiciels")

ID 276

32. Quel(s) application(s) ou logiciel(s) avez-vous utilisé(s)? *

- [ ] Garage Band
- [ ] Audacity
- [ ] Prezi
- [ ] Power Point
- [ ] Abbleton
- [ ] Autre

*

LOGIC Hidden unless: #27 Question "Quelles sont les ressources pédagogiques que vous avez utilisées pendant la pandémie ?" is one of the following answers ("Hygiène et mesures sanitaires")

ID 277

33. Quel(s) type(s) de mesures sanitaire avez-vous utilisé(s) comme référence? *

- [ ] Publications du gouvernement
- [ ] Publications d'associations professionnelles
- [ ] Articles scientifiques
- [ ] Autre

*

ID 166

34. Si vous désirez laisser un commentaire au sujet des outils technologiques durant la pandémie, veuillez l'inscrire ici.

SECTION C Printemps 2020 : Réouverture des écoles (à distance ou en présence)

LOGIC Show/hide trigger exists.

ID 23

35. Comment s’est poursuivi votre enseignement suite à la réouverture des écoles? *

- ○ Mon école a été rouverte et mon enseignement s'est poursuivi en présentiel
- ○ Mon école a été rouverte et mon enseignement s'est poursuivi en présentiel ET à distance
- ○ Mon école n'a pas été rouverte et mon enseignement s'est poursuivi à distance exclusivement
- ○ J'ai été réaffecté à d'autres tâches
- ○ Je n'ai pas enseigné et je n'ai pas été réaffecté à d'autres tâches dans mon école

SECTION D Printemps 2020 : Enseignement en présence

LOGIC Hidden unless: #35 Question "Comment s’est poursuivi votre enseignement suite à la réouverture des écoles?" is one of the following answers ("Mon école a été rouverte et mon enseignement s'est poursuivi en présentiel")

ID 25

36. Quels changements dans les lieux physiques ont étés apportés pour l'enseignement de la musique ? *

- ☐ Aucun
- ☐ Diminution du nombre d'élèves par classe
- ☐ Musique dans la classe du titulaire
- ☐ Autre (précisez) ________ *

LOGIC Hidden unless: #35 Question "Comment s’est poursuivi votre enseignement suite à la réouverture des écoles?" is one of the following answers ("Mon école a été rouverte et mon enseignement s'est poursuivi en présentiel")

ID 26

37. Quel équipement sanitaire avez-vous dû porter? *

- ☐ Visière
- ☐ Lunette
- ☐ Masque
- ☐ Autre (précisez) *

LOGIC Hidden unless: #35 Question "Comment s’est poursuivi votre enseignement suite à la réouverture des écoles?" is one of the following answers ("Mon école a été rouverte et mon enseignement s'est poursuivi en présentiel")

ID 27

38. Quelles pratiques musicales avez-vous continuée, modifiées ou cessées ?

	Poursuivie tel quel	Modifiée	Cessée	Ne s'applique pas
Chant	○	○	○	○
Instrumentarium Orff	○	○	○	○
Mouvement	○	○	○	○
Flute à bec	○	○	○	○
Jeux musicaux de groupe	○	○	○	○
Ukulélé	○	○	○	○
Guitare	○	○	○	○
Instrument à vent / orchestre harmonie	○	○	○	○
Stage band	○	○	○	○
Orchestre à cordes	○	○	○	○
Comédie musicale	○	○	○	○
Bands pop	○	○	○	○
Orchestres symphonique	○	○	○	○
Percussions (Djembé, drumline et autres)	○	○	○	○

LOGIC Hidden unless: #35 Question "Comment s’est poursuivi votre enseignement suite à la réouverture des écoles?" is one of the following answers ("Mon école a été rouverte et mon enseignement s'est poursuivi en présentiel")

ID 45

39. Voulez-vous nous donner plus de précisions au sujet des activités musicales ? (optionnel)

LOGIC Hidden unless: #35 Question "Comment s’est poursuivi votre enseignement suite à la réouverture des écoles?" is one of the following answers ("Mon école a été rouverte et mon enseignement s'est poursuivi en présentiel")

ID 46

40. Avez-vous réussi à poursuivre votre planification globale prévue? *

- Pas du tout
- Un peu
- Presqu'entièrement
- Entièrement

LOGIC Hidden unless: #35 Question "Comment s’est poursuivi votre enseignement suite à la réouverture des écoles?" is one of the following answers ("Mon école a été rouverte et mon enseignement s'est poursuivi en présentiel")

ID 47

41. Combien de temps avez-vous dû consacrer à votre préparation? *

- Moins que d'habitude
- Comme d'habitude
- Un peu plus
- Beaucoup plus

LOGIC Hidden unless: #35 Question "Comment s’est poursuivi votre enseignement suite à la réouverture des écoles?" is one of the following answers ("Mon école a été rouverte et mon enseignement s'est poursuivi en présentiel")

ID 143

42. Quelle(s) sont les compétence(s) que vous avez abordé(s)? *

- Interpréter
- Apprécier
- Créer/Inventer
- Aucune, merci de préciser

LOGIC Hidden unless: #35 Question "Comment s’est poursuivi votre enseignement suite à la réouverture des écoles?" is one of the following answers ("Mon école a été rouverte et mon enseignement s'est poursuivi en présentiel")

ID 167

43. Quelle(s) sont les compétence(s) pour lesquelles vous avez donné des devoirs? *

- [ ] Interpréter
- [ ] Apprécier
- [ ] Créer/Inventer
- [ ] Aucune, merci de préciser

LOGIC Hidden unless: #35 Question "Comment s’est poursuivi votre enseignement suite à la réouverture des écoles?" is one of the following answers ("Mon école a été rouverte et mon enseignement s'est poursuivi en présentiel")

ID 142

44. Quelle(s) sont les compétence(s) que vous avez évaluée(s)? *

- [ ] Interpréter
- [ ] Apprécier
- [ ] Créer/Inventer
- [ ] Aucune, merci de préciser

LOGIC Hidden unless: #35 Question "Comment s’est poursuivi votre enseignement suite à la réouverture des écoles?" is one of the following answers ("Mon école a été rouverte et mon enseignement s'est poursuivi en présentiel")

ID 51

45. Comment était la motivation des élèves en général ? *

- Beaucoup plus faible que d'habitude
- Plus faible que d'habitude
- Comme d'habitude
- Plus élevée que d'habitude
- Beaucoup plus élevée de que d'habitude

LOGIC Hidden unless: #35 Question "Comment s’est poursuivi votre enseignement suite à la réouverture des écoles?" is one of the following answers ("Mon école a été rouverte et mon enseignement s'est poursuivi en présentiel")

ID 53

46. Comment était votre motivation en général ? *

- Beaucoup plus faible que d'habitude
- Plus faible que d'habitude
- Comme d'habitude
- Plus élevée que d'habitude
- Beaucoup plus élevée de que d'habitude

LOGIC Hidden unless: #35 Question "Comment s’est poursuivi votre enseignement suite à la réouverture des écoles?" is one of the following answers ("Mon école a été rouverte et mon enseignement s'est poursuivi en présentiel")

ID 54

47. Si votre enseignement était exclusivement en présence, avez-vous aussi utilisé une ou des plateforme.s électronique.s? Si oui, précisez laquelle. *

- ○ Oui (précisez)
- ○ Non

SECTION E Printemps 2020: Enseignement en présence et à distance

LOGIC Hidden unless: #35 Question "Comment s’est poursuivi votre enseignement suite à la réouverture des écoles?" is one of the following answers ("Mon école a été rouverte et mon enseignement s'est poursuivi en présentiel ET à distance")

ID 226

48. Vers quelle(s) ressource(s) vous-êtes-vous tournée(s) pour vous aider dans ce virage vers l’enseignement à distance? *

- ☐ Pistes pédagogiques du Ministère de l'Éducation
- ☐ Capsules télévisuelles de Télé-Québec
- ☐ Conseillers pédagogiques
- ☐ Enseignants généralistes
- ☐ Autres enseignants en musique
- ☐ Sites Internet
- ☐ Associations professionnelles
- ☐ Autre (préciser) *
- ☐ Aucun

LOGIC Hidden unless: #35 Question "Comment s’est poursuivi votre enseignement suite à la réouverture des écoles?" is one of the following answers ("Mon école a été rouverte et mon enseignement s'est poursuivi en présentiel ET à distance")

ID 175

49. Quels changements dans les lieux physiques ont été apportés pour l’enseignement de la musique EN PRÉSENCE ? *

- [ ] Aucun
- [ ] Diminution du nombre d'élèves par classe
- [ ] Musique dans la classe du titulaire
- [ ] Autre (précisez) *

LOGIC Hidden unless: #35 Question "Comment s’est poursuivi votre enseignement suite à la réouverture des écoles?" is one of the following answers ("Mon école a été rouverte et mon enseignement s'est poursuivi en présentiel ET à distance")

ID 176

50. Quel équipement sanitaire avez-vous dû porter ? *

- [ ] Visière
- [ ] Lunettes
- [ ] Masque
- [ ] Autre (précisez) *

LOGIC Hidden unless: #35 Question "Comment s’est poursuivi votre enseignement suite à la réouverture des écoles?" is one of the following answers ("Mon école a été rouverte et mon enseignement s'est poursuivi en présentiel ET à distance")

ID 177

51. Quel moyen avez-vous utilisé pour interagir avec vos élèves ? *

- [ ] Teams
- [ ] Zoom
- [ ] Facebook Live
- [ ] Skype
- [ ] Google Classroom
- [ ] Autre (précisez) *
- [ ] Aucun

LOGIC Hidden unless: #35 Question "Comment s’est poursuivi votre enseignement suite à la réouverture des écoles?" is one of the following answers ("Mon école a été rouverte et mon enseignement s'est poursuivi en présentiel ET à distance")

ID 178

52. À quelle fréquence avez-vous rencontré vos élèves à distance ? *

- ( ) Moins d'une fois par semaine
- ( ) Une à deux fois par semaine
- ( ) Trois fois et plus par semaine
- ( ) Ne s'applique pas

LOGIC Hidden unless: #35 Question "Comment s’est poursuivi votre enseignement suite à la réouverture des écoles?" is one of the following answers ("Mon école a été rouverte et mon enseignement s'est poursuivi en présentiel ET à distance")

ID 179

53. À quel pourcentage évalueriez-vous la présence de vos élèves lors des rencontres en ligne ? *

- Une seule rencontre
- 25 % des rencontres
- 50 % des rencontres
- 80 % des rencontres
- 100 % des rencontres
- Autre (préciser)
- Ne s'applique pas

LOGIC Hidden unless: #35 Question "Comment s’est poursuivi votre enseignement suite à la réouverture des écoles?" is one of the following answers ("Mon école a été rouverte et mon enseignement s'est poursuivi en présentiel ET à distance")

ID 180

54. Quel est le taux de participation de vos élèves aux activités pédagogiques proposées ? *

- 0% à 10%
- 25 %
- 50 %
- 80 %
- 100 %
- Ne s'applique pas

LOGIC Hidden unless: #35 Question "Comment s’est poursuivi votre enseignement suite à la réouverture des écoles?" is one of the following answers ("Mon école a été rouverte et mon enseignement s'est poursuivi en présentiel ET à distance")

ID 181

55. Est-ce qu’un parent (ou tuteur, gardien) était présent en ligne avec les élèves ? *

- Jamais
- Parfois
- La plupart du temps
- Tout le temps
- Autre (préciser)
- Ne s'applique pas

LOGIC Hidden unless: #35 Question "Comment s’est poursuivi votre enseignement suite à la réouverture des écoles?" is one of the following answers ("Mon école a été rouverte et mon enseignement s'est poursuivi en présentiel ET à distance")

ID 182

56. À quelle fréquence avez-vous déposé du matériel pédagogique en ligne pour vos élèves ? *

- Moins d'une fois par semaine
- 1 à 2 fois par semaine
- 3 à 5 fois par semaine
- 7 à 10 fois par semaine
- 11 fois et plus par semaine
- Ne s'applique pas

LOGIC Hidden unless: #35 Question "Comment s’est poursuivi votre enseignement suite à la réouverture des écoles?" is one of the following answers ("Mon école a été rouverte et mon enseignement s'est poursuivi en présentiel ET à distance")

ID 183

57. Avez-vous réussi à poursuivre votre planification globale prévue ? *

- Pas du tout
- Un peu
- Presque entièrement
- Entièrement

LOGIC Hidden unless: #35 Question "Comment s’est poursuivi votre enseignement suite à la réouverture des écoles?" is one of the following answers ("Mon école a été rouverte et mon enseignement s'est poursuivi en présentiel ET à distance")

ID 184

58. Combien de temps avez-vous dû consacrer à votre préparation ? *

- Beaucoup moins que d'habitude
- Moins que d'habitude
- Comme d'habitude
- Un peu plus que d'habitude
- Beaucoup plus que d'habitude

LOGIC Hidden unless: #35 Question "Comment s’est poursuivi votre enseignement suite à la réouverture des écoles?" is one of the following answers ("Mon école a été rouverte et mon enseignement s'est poursuivi en présentiel ET à distance")

ID 185

59. Quelles sont les compétences que vous avez abordées ? *

- [ ] Interpréter
- [ ] Apprécier
- [ ] Créer/inventer
- [ ] Aucune, merci de préciser pourquoi

LOGIC Hidden unless: #35 Question "Comment s’est poursuivi votre enseignement suite à la réouverture des écoles?" is one of the following answers ("Mon école a été rouverte et mon enseignement s'est poursuivi en présentiel ET à distance")

ID 186

60. Quelles sont les compétences pour lesquelles vous avez donné des devoirs ? *

- [ ] Interpréter
- [ ] Apprécier
- [ ] Créer/inventer
- [ ] Aucune, merci de préciser pourquoi

LOGIC Hidden unless: #35 Question "Comment s’est poursuivi votre enseignement suite à la réouverture des écoles?" is one of the following answers ("Mon école a été rouverte et mon enseignement s'est poursuivi en présentiel ET à distance")

ID 187

61. Quelles sont les compétences que vous avez évaluées *

- [ ] Interpréter
- [ ] Apprécier
- [ ] Créer/inventer
- [ ] Aucune, merci de préciser pourquoi

LOGIC Hidden unless: #35 Question "Comment s’est poursuivi votre enseignement suite à la réouverture des écoles?" is one of the following answers ("Mon école a été rouverte et mon enseignement s'est poursuivi en présentiel ET à distance")

ID 188

62. Comment était la motivation des élèves en général ? *

- ○ Beaucoup plus faible que d'habitude
- ○ Plus faible que d'habitude
- ○ Comme d'habitude
- ○ Plus que d'habitude
- ○ Beaucoup plus que d'habitude

LOGIC Hidden unless: #35 Question "Comment s’est poursuivi votre enseignement suite à la réouverture des écoles?" is one of the following answers ("Mon école a été rouverte et mon enseignement s'est poursuivi en présentiel ET à distance")

ID 189

63. Comment était votre motivation en général ? *

- Beaucoup plus faible que d'habitude
- Plus faible que d'habitude
- Comme d'habitude
- Plus que d'habitude
- Beaucoup plus que d'habitude

LOGIC Hidden unless: #35 Question "Comment s’est poursuivi votre enseignement suite à la réouverture des écoles?" is one of the following answers ("Mon école a été rouverte et mon enseignement s'est poursuivi en présentiel ET à distance")

ID 190

64. Comment décriviez-vous vos cours à distance ? *

- ☐ Interactif
- ☐ Magistral
- ☐ Par projet
- ☐ Recherche individuelle
- ☐ Classe inversée
- ☐ Recherche en équipe
- ☐ Discussions
- ☐ Production artistique
- ☐ Écoute et questionnaire
- ☐ Pratique instrumentale individuelle
- ☐ Quizz
- ☐ Autres (préciser) ______ *

LOGIC Hidden unless: #35 Question "Comment s’est poursuivi votre enseignement suite à la réouverture des écoles?" is one of the following answers ("Mon école a été rouverte et mon enseignement s'est poursuivi en présentiel ET à distance")

ID 324

65. Quelles pratiques musicales avez-vous continuées, modifiées ou cessées ?   (Cochez les réponses pertinentes)

	Poursuivie tel quel	Modifiée	Cessée	Ne s'applique pas
Chant	○	○	○	○
Instrumentarium Orff	○	○	○	○
Mouvement	○	○	○	○
Flute à bec	○	○	○	○
Jeux musicaux de groupe	○	○	○	○
Ukulélé	○	○	○	○
Guitare	○	○	○	○
Instrument à vent / orchestre harmonie	○	○	○	○
Stage band	○	○	○	○
Orchestre à cordes	○	○	○	○
Comédie musicale	○	○	○	○
Bands pop	○	○	○	○
Orchestres symphonique	○	○	○	○
Percussions (Djembé, drumline et autres)	○	○	○	○

LOGIC Hidden unless: #35 Question "Comment s’est poursuivi votre enseignement suite à la réouverture des écoles?" is one of the following answers ("Mon école a été rouverte et mon enseignement s'est poursuivi en présentiel ET à distance")

ID 209

66. Voulez-vous nous donner plus de précisions (optionnel) ?

LOGIC Hidden unless: #35 Question "Comment s’est poursuivi votre enseignement suite à la réouverture des écoles?" is one of the following answers ("Mon école a été rouverte et mon enseignement s'est poursuivi en présentiel ET à distance")

ID 224

67. Si vous avez créé du matériel pédagogique en ligne pour vos élèves, nous aimerions y avoir accès de façon à analyser les pratiques enseignantes. Toutes les données seront anonymisées et utilisées pour les seules fins de cette recherche. Pourriez-vous nous donner un accès à votre matériel soit vers un partage par Drive ou vers un lien URL (You tube, site personnel, etc.) ? *

- Oui
- Non

SECTION F Printemps 2020 : Enseignement à distance exclusivement

LOGIC Hidden unless: #35 Question "Comment s’est poursuivi votre enseignement suite à la réouverture des écoles?" is one of the following answers ("Mon école n'a pas été rouverte et mon enseignement s'est poursuivi à distance exclusivement")

ID 289

68. Vers quelle(s) ressource(s) vous-êtes-vous tournée(s) pour vous aider dans ce virage vers l’enseignement à distance? *

- [ ] Pistes pédagogiques du Ministère de l'Éducation
- [ ] Capsules Télé-Québec
- [ ] Conseillers pédagogiques
- [ ] Enseignants généralistes
- [ ] Autres enseignants en musique
- [ ] Site Internet
- [ ] Association professionnelle
- [ ] Autre (préciser)
- [ ] Aucun

LOGIC Hidden unless: #35 Question "Comment s’est poursuivi votre enseignement suite à la réouverture des écoles?" is one of the following answers ("Mon école n'a pas été rouverte et mon enseignement s'est poursuivi à distance exclusivement")

ID 290

69. Quel(s) moyen(s) avez-vous utilisé pour communiquer avec vos élèves/parents? *

- [ ] Courrier
- [ ] Téléphone
- [ ] Courriel
- [ ] Facebook
- [ ] Autre (préciser) *

LOGIC Hidden unless: #35 Question "Comment s’est poursuivi votre enseignement suite à la réouverture des écoles?" is one of the following answers ("Mon école n'a pas été rouverte et mon enseignement s'est poursuivi à distance exclusivement")

ID 292

70. Quel moyen avez-vous utilisé pour interagir avec vos élèves? *

- [ ] Teams
- [ ] Zoom
- [ ] Facebook Live
- [ ] Skype
- [ ] Autre (préciser) *
- [ ] Aucun

LOGIC Hidden unless: #35 Question "Comment s’est poursuivi votre enseignement suite à la réouverture des écoles?" is one of the following answers ("Mon école n'a pas été rouverte et mon enseignement s'est poursuivi à distance exclusivement")

ID 59

71. À quelle fréquence avez-vous rencontré vos élèves à distance ? *

- Moins d'une fois par semaine
- Une à deux fois par semaine
- Trois fois et plus par semaine
- Ne s'applique pas

LOGIC Hidden unless: #35 Question "Comment s’est poursuivi votre enseignement suite à la réouverture des écoles?" is one of the following answers ("Mon école n'a pas été rouverte et mon enseignement s'est poursuivi à distance exclusivement")

ID 171

72. À quel pourcentage évalueriez-vous la présence des vos élèves lors des rencontres en ligne? *

- Une seule rencontre
- 25% des rencontres
- 50% des rencontres
- 80% des rencontres
- 100% des rencontres
- Ne s'applique pas

LOGIC Hidden unless: #35 Question "Comment s’est poursuivi votre enseignement suite à la réouverture des écoles?" is one of the following answers ("Mon école n'a pas été rouverte et mon enseignement s'est poursuivi à distance exclusivement")

ID 60

73. Quel est le taux de participation de vos élèves aux activité pédagogiques proposées :
Nombre d'élèves à chaque rencontre *

- 0 à 10%
- 25%
- 50%
- 80%
- 100%
- Ne s'applique pas

LOGIC Hidden unless: #35 Question "Comment s’est poursuivi votre enseignement suite à la réouverture des écoles?" is one of the following answers ("Mon école n'a pas été rouverte et mon enseignement s'est poursuivi à distance exclusivement")

ID 62

74. Est-ce qu’un parent (ou tuteur, gardien) était présent en ligne avec les élèves ? *

- Jamais
- Parfois
- La plupart du temps
- Tout le temps
- Autre (Précisez)
- Ne s'applique pas

LOGIC Hidden unless: #35 Question "Comment s’est poursuivi votre enseignement suite à la réouverture des écoles?" is one of the following answers ("Mon école n'a pas été rouverte et mon enseignement s'est poursuivi à distance exclusivement")

ID 64

75. À quelle fréquence avez-vous déposé du matériel pédagogique en ligne pour vos élèves (par semaine)? *

- Moins d'une fois par semaine
- 1 à 2 fois
- 3 à 5 fois
- 7 à 10 fois
- 11 fois et plus

LOGIC Hidden unless: #35 Question "Comment s’est poursuivi votre enseignement suite à la réouverture des écoles?" is one of the following answers ("Mon école n'a pas été rouverte et mon enseignement s'est poursuivi à distance exclusivement")

ID 68

76. Avez-vous réussi à poursuivre votre planification globale prévue? *

- Pas du tout
- Un peu
- Presque entièrement
- Entièrement

LOGIC Hidden unless: #35 Question "Comment s’est poursuivi votre enseignement suite à la réouverture des écoles?" is one of the following answers ("Mon école n'a pas été rouverte et mon enseignement s'est poursuivi à distance exclusivement")

ID 69

77. Combien de temps avez-vous dû consacrer à votre préparation ? *

- Beaucoup moins que d'habitude
- Moins que d'habitude
- Comme d'habitude
- Plus que d'habitude
- Beaucoup plus que d'habitude

LOGIC Hidden unless: #35 Question "Comment s’est poursuivi votre enseignement suite à la réouverture des écoles?" is one of the following answers ("Mon école n'a pas été rouverte et mon enseignement s'est poursuivi à distance exclusivement")

ID 147

78. Quelles sont les compétences que vous avez abordées? *

- [ ] Interpréter
- [ ] Apprécier
- [ ] Créer/Inventer
- [ ] Aucune, merci de préciser pourquoi

LOGIC Hidden unless: #35 Question "Comment s’est poursuivi votre enseignement suite à la réouverture des écoles?" is one of the following answers ("Mon école n'a pas été rouverte et mon enseignement s'est poursuivi à distance exclusivement")

ID 168

79. Quels sont les compétences pour lesquelles vous avez donné des devoirs? *

- [ ] Interpréter
- [ ] Apprécier
- [ ] Créer/Inventer
- [ ] Aucune, merci de préciser pourquoi

LOGIC Hidden unless: #35 Question "Comment s’est poursuivi votre enseignement suite à la réouverture des écoles?" is one of the following answers ("Mon école n'a pas été rouverte et mon enseignement s'est poursuivi à distance exclusivement")

ID 146

80. Quelles sont les compétences que vous avez évaluées? *

- [ ] Interpréter
- [ ] Apprécier
- [ ] Créer/Inventer
- [ ] Aucune, merci de préciser pourquoi

LOGIC Hidden unless: #35 Question "Comment s’est poursuivi votre enseignement suite à la réouverture des écoles?" is one of the following answers ("Mon école n'a pas été rouverte et mon enseignement s'est poursuivi à distance exclusivement")

ID 76

81. Comment était la motivation des élèves en général ? *

- Beaucoup plus faible que d’habitude
- Plus faible que d’habitude
- Comme d’habitude
- Plus élevée que d’habitude
- Beaucoup plus élevée que d’habitude

LOGIC Hidden unless: #35 Question "Comment s’est poursuivi votre enseignement suite à la réouverture des écoles?" is one of the following answers ("Mon école n'a pas été rouverte et mon enseignement s'est poursuivi à distance exclusivement")

ID 79

82. Comment était votre motivation en général ? *

- Beaucoup plus faible que d’habitude
- Plus faible que d’habitude
- Comme d’habitude
- Plus élevée que d’habitude
- Beaucoup plus élevée que d’habitude

LOGIC Hidden unless: #35 Question "Comment s’est poursuivi votre enseignement suite à la réouverture des écoles?" is one of the following answers ("Mon école n'a pas été rouverte et mon enseignement s'est poursuivi à distance exclusivement")

ID 80

83. Comment décriviez-vous vos cours à distance? *

- [ ] Interactif
- [ ] Magistral
- [ ] Par projet
- [ ] Recherche individuelle
- [ ] Classe inversée
- [ ] Recherche en équipe
- [ ] Discussions
- [ ] Production artistique
- [ ] Écoute et questionnaire
- [ ] Pratique instrumentale individuelle
- [ ] Quizz
- [ ] Autres (précisez)

*

LOGIC Hidden unless: #35 Question "Comment s’est poursuivi votre enseignement suite à la réouverture des écoles?" is one of the following answers ("Mon école n'a pas été rouverte et mon enseignement s'est poursuivi à distance exclusivement")

ID 294

84. Quelles pratiques musicales avez-vous continuées, modifiées ou cessées ?   (Cochez les réponses pertinentes)

	Poursuivie tel quel	Modifiée	Cessée	Ne s'applique pas
Chant	○	○	○	○
Instrumentarium Orff	○	○	○	○
Mouvement	○	○	○	○
Flute à bec	○	○	○	○
Jeux musicaux de groupe	○	○	○	○
Ukulélé	○	○	○	○
Guitare	○	○	○	○
Instrument à vent / orchestre harmonie	○	○	○	○
Stage band	○	○	○	○
Orchestre à cordes	○	○	○	○
Comédie musicale	○	○	○	○
Bands pop	○	○	○	○
Orchestres symphonique	○	○	○	○
Percussions (Djembé, drumline et autres)	○	○	○	○

LOGIC Hidden unless: #35 Question "Comment s’est poursuivi votre enseignement suite à la réouverture des écoles?" is one of the following answers ("Mon école n'a pas été rouverte et mon enseignement s'est poursuivi à distance exclusivement")

ID 98

85. Voulez-vous nous donner plus de précisions au sujet des activités musicales?

LOGIC Hidden unless: #35 Question "Comment s’est poursuivi votre enseignement suite à la réouverture des écoles?" is one of the following answers ("Mon école n'a pas été rouverte et mon enseignement s'est poursuivi à distance exclusivement")

ID 106

86. Si vous avez créé du matériel pédagogique en ligne pour vos élèves, nous aimerions y avoir accès de façon à analyser les pratiques enseignantes. Toutes les données seront anonymisées et utilisé pour les seules fins de cette recherche. Pourriez-vous nous donner un accès à votre matériel soit vers un partage par Drive ou vers un lien URL (you tube, site personnel, ect…)? *

- ○ Oui
- ○ Non

SECTION G Printemps 2020 : Si vous avez été réaffecté à d'autre tâches

LOGIC Hidden unless: #35 Question "Comment s’est poursuivi votre enseignement suite à la réouverture des écoles?" is one of the following answers ("J'ai été réaffecté à d'autres tâches","Je n'ai pas enseigné et je n'ai pas été réaffecté à d'autres tâches dans mon école")

ID 172

87. En quoi vos tâches ont-elles changées? *

- Je suis passé du secondaire au primaire
- J’ai enseigné une autre discipline :
- Je suis devenu titulaire de classe :
- Autres (préciser)

LOGIC Hidden unless: #35 Question "Comment s’est poursuivi votre enseignement suite à la réouverture des écoles?" is one of the following answers ("J'ai été réaffecté à d'autres tâches")

ID 173

88. Pour quelle(s) raison(s) vos tâches ont-elle changé? *

- Manque de personnel.
- Augmentation du nombre de classes
- Arrêt des cours de musique pour des raisons sanitaires
- Arrêt des cours de musique par manque d’espace
- Autres (préciser)

LOGIC Hidden unless: #35 Question "Comment s’est poursuivi votre enseignement suite à la réouverture des écoles?" is one of the following answers ("J'ai été réaffecté à d'autres tâches")

ID 174

89. Si vous le souhaitez, dites-nous quels défis ces changements ont-ils représentés

LOGIC Hidden unless: #35 Question "Comment s’est poursuivi votre enseignement suite à la réouverture des écoles?" is one of the following answers ("J'ai été réaffecté à d'autres tâches")

ID 109

90. Combien de temps avez-vous dû consacrer à votre préparation? *

- Beaucoup moins que d’habitude
- Moins que d’habitude
- Comme d’habitude
- Plus que d’habitude
- Beaucoup plus que d’habitude

LOGIC Hidden unless: #35 Question "Comment s’est poursuivi votre enseignement suite à la réouverture des écoles?" is one of the following answers ("J'ai été réaffecté à d'autres tâches")

ID 110

91. Comment était votre motivation en général ? *

- Beaucoup plus faible que d’habitude
- Plus faible que d’habitude
- Comme d’habitude
- Plus élevée que d’habitude
- Beaucoup plus élevée que d’habitude

SECTION H Rentrée automne 2020

Page exit logic: Skip / Disqualify Logic
IF: #92 Question "Cet automne, avez-vous conserver une tâche d’enseignement similaire à celle de l’année scolaire 2019-2020?" is one of the following answers ("Non applicable, préciser pourquoi (ex. congé maternité ou maladie, sabbatique, retraite, etc.)") **THEN:** Jump to page 11 - SECTION I Désirez-vous nous communiquer d'autres informations?

LOGIC Show/hide trigger exists.

ID 115

92. Cet automne, avez-vous conserver une tâche d’enseignement similaire à celle de l’année scolaire 2019-2020? *

- Oui
- Non
- Non applicable, préciser pourquoi (ex. congé maternité ou maladie, sabbatique, retraite, etc.)

SECTION H Rentrée automne 2020

LOGIC Hidden unless: #92 Question "Cet automne, avez-vous conserver une tâche d'enseignement similaire à celle de l'année scolaire 2019-2020?" is one of the following answers ("Non")

ID 112

93. Quel changement a été apporté dans vos tâches? *

- Je suis passé du secondaire au primaire
- Je suis devenu titulaire de classe
- J'enseigne une autre discipline
- Autre

LOGIC Hidden unless: #92 Question "Cet automne, avez-vous conserver une tâche d'enseignement similaire à celle de l'année scolaire 2019-2020?" is one of the following answers ("Non")

ID 113

94. Pour quelle(s) raison(s) ces changements ont eu lieu? *

- [ ] Manque de personnel
- [ ] Besoin de combler ma tâche
- [ ] Contrainte dû à l'organisation des groupes-classe
- [ ] Arrêt des cours de musique pour des raisons sanitaires
- [ ] Arrêt des cours de musique par manque d'espace
- [ ] Autre (précisez)

*

LOGIC Hidden unless: #92 Question "Cet automne, avez-vous conserver une tâche d’enseignement similaire à celle de l’année scolaire 2019-2020?" is one of the following answers ("Non")

ID 116

95. Quels sont les principaux changements entre votre tâche d'enseignement 2019-2020 et 2020-2021 (Instruments enseignés, Locaux attribués, etc.)? *

ID 117

96. Comment se poursuit votre enseignement cet automne? *

- Mon école a été rouverte et mon enseignement s’est poursuivi en présentiel
- Mon école a été rouverte et mon enseignement s’est poursuivi en présentiel ET en ligne
- Mon école n’a pas été rouverte et mon enseignement s’est poursuivi en ligne

ID 118

97. Combien de temps avez-vous dû consacrer à votre préparation? *

- Beaucoup moins que d’habitude
- Moins que d’habitude
- Comme d’habitude
- Plus que d’habitude
- Beaucoup plus que d’habitude

ID 119

98. Comment est votre motivation en général cet automne? *

- Beaucoup plus faible que d’habitude
- Plus faible que d’habitude
- Comme d’habitude
- Plus élevée que d’habitude
- Beaucoup plus élevée que d’habitude

ID 120

99. Quelles pratiques musicales avez-vous continuées, modifiées ou cessées ? (Cochez les réponses pertinentes)

	Poursuivie tel quel	Modifiée	Cessée	Ne s'applique pas
Chant	○	○	○	○
Instrumentarium Orff	○	○	○	○
Mouvement	○	○	○	○
Flute à bec	○	○	○	○
Jeux musicaux de groupe	○	○	○	○
Ukulélé	○	○	○	○
Guitare	○	○	○	○
Instrument à vent / orchestre harmonie	○	○	○	○
Stage band	○	○	○	○
Orchestre à cordes	○	○	○	○
Comédie musicale	○	○	○	○
Bands pop	○	○	○	○
Orchestres symphonique	○	○	○	○
Percussions (Djembé, drumline et autres)	○	○	○	○

ID 138

100. Voulez-vous nous donner plus de précisions (optionnel)?

SECTION I Désirez-vous nous communiquer d'autres informations?

ID 139

101. Écrivez-nous les informations que vous souhaitez nous communiquer

LOGIC Show/hide trigger exists.

ID 269

102. Accepteriez-vous de participer à une entrevue pour donner plus d’information ?

- Non
- Oui

LOGIC Hidden unless: #102 Question "Accepteriez-vous de participer à une entrevue pour donner plus d’information ?" is one of the following answers ("Oui")

ID 339

103. Merci de nous donner vos coordonnées pour que nous puissions vous joindre.

LOGIC Hidden unless: QUESTION NOT FOUND! is exactly equal to [NO OPTIONS SET]

ID 283

104. Nous respectons votre choix de ne pas participer au sondage. Au revoir!

S'il s'agissait d'une erreur, nous vous invitons à retourner à la page du consentement éthique
